# Hardware considerations in infection and nonunion management: When and how to revise the fixation

**DOI:** 10.1097/OI9.0000000000000055

**Published:** 2020-03-23

**Authors:** Graeme Hoit, Marissa Bonyun, Aaron Nauth

**Affiliations:** a Division of Orthopaedic Surgery, Department of Surgery, University of Toronto; b Department of Orthopaedic Surgery, St. Michaels Hospital, Toronto, ON, Canada

**Keywords:** broken hardware, fracture healing, fracture nonunion, fracture-related infection, fracture revision, hardware removal, infection, metabolic causes of nonunion, nonunion, orthopaedic trauma

## Abstract

The occurrence of both nonunion and fracture-related infection provides challenges for both the patient and the treating orthopaedic surgeon, with the potential need for complex reconstructive procedures to achieve union and/or eradicate infection. In addition to addressing the multiple different factors that often contribute to nonunion, surgeons are often forced to deal with difficult hardware issues at the time of revision surgery including infected hardware, loose or failing hardware, malaligned hardware, or inappropriate hardware constructs. This article reviews common causes of nonunions with emphasis on infection management and provides indications and techniques for hardware removal in the context of an algorithmic approach to nonunion management with illustrative case examples.

## 1 Introduction

The majority of surgically treated fractures go on to adequate bony union. However, 5% to 10% of these fractures fail to heal and develop a nonunion.^[1–4]^ Nonunion is defined as failure of bony healing by 9 months, with no interval progression of radiographic healing over the last 3 months.^[5]^ The occurrence of nonunion provides challenges for both the patient and the treating orthopaedic surgeon, with the potential need for complex reconstructive procedures to achieve union. It is important to recognize the effects that acute and chronic fracture-related infection (FRI) can have on the ability to achieve bony union in a trauma setting.

Due to the demographics of orthopaedic trauma patients, nonunions often affect young individuals, most commonly between the ages of 35 and 44,^[1]^ with substantial implications on their physical, financial and psychological well-being.^[6]^ This has significant individual, as well as societal impact, as these patients are typically in their most economically productive years. In addition, the impact on quality of life is substantial. Brinker et al^[7]^ examined quality of life measures in patients living with a tibial nonunion and demonstrated that their reported quality of life was poorer than patients who were postmyocardial infarction or stroke. Nonunions have also been shown to drastically increase the amount and strength of opioids consumed in orthopaedic trauma patients, a growing concern in the context of the current opioid epidemic.^[8]^ Additionally, nonunions pose a significant cost to healthcare systems. Estimates of direct healthcare costs as a result of a single nonunion have ranged from 11,000 to 100,000 USD more than a fracture that heals.^[1,8–13]^

The significant societal and personal costs of nonunion care are often exacerbated by the complexities of their surgical management. In addition to addressing the multiple different factors that often contribute to nonunion, surgeons are often forced to deal with difficult hardware issues at the time of revision surgery including infected hardware, loose or failing hardware, malaligned hardware, inappropriate hardware constructs, or peri-implant fractures. The purpose of this article is to highlight the role of surgical infection in the development of nonunions and provide indications and techniques for hardware removal in the context of an algorithmic approach to nonunion management.

## 2 Section 1: principles of nonunion management

The occurrence of nonunion is frequently multifactorial and several potential contributing factors should be considered including infection, mechanical, biological, metabolic, and patient factors (see Table 1).^[14]^ Mills et al^[15]^ examined 100 patients with nonunions and determined that 69% of them had more than 1 contributing cause, with peri-implant infection present in 38% of cases. The successful management of nonunion requires an algorithmic approach to identifying and treating all potential contributing factors, and often requires that multiple factors are addressed sequentially or simultaneously (see Table 1).

**Table 1:**
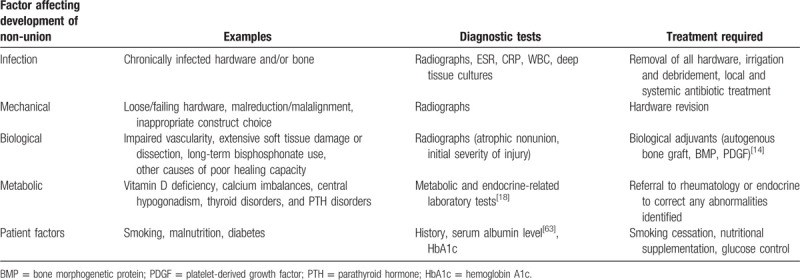
Multifactorial approach to fracture nonunion management

Prior to surgical intervention, factors that can be optimized to assist in healing should be identified and, when possible, mitigated or minimized. Smoking reduction, diabetes control, nutritional optimization are all important interventions that can play a significant role in fracture healing and immune response.^[14,16,17]^ Additionally, metabolic causes for nonunion should be considered in particular in patients with multiple low energy fractures developing nonunion, or a nonunion of a pubic rami or sacral ala fracture.^[18]^ Evidence suggests that a multidisciplinary approach to metabolic nonunions leads to superior results with involvement of endocrinology for appropriate workup and treatment.^[19]^ Abnormalities in vitamin D, calcium, parathyroid hormone, thyroid function, and growth hormone have all been commonly linked to impaired fracture healing.^[14]^ Unlike in infectious cases, metabolic nonunions may not require removal of hardware, but rather should be treated with goals of promoting bone healing. In a cohort of 31 patients with nonunions, Brinker et al^[18]^ demonstrated metabolic treatment alone can at times be sufficient, with 8 patients achieving union despite no surgical intervention.

## 3 Section 2: revising and removing hardware

Within the context of an algorithmic and principle-based approach to nonunion there are several indications for hardware removal and/or revision including:

1. Infected hardware
2. Loose or failing hardware
3. Malaligned hardware
4. Inappropriate hardware construct

### 3.1 Infection

**Figure 1:**
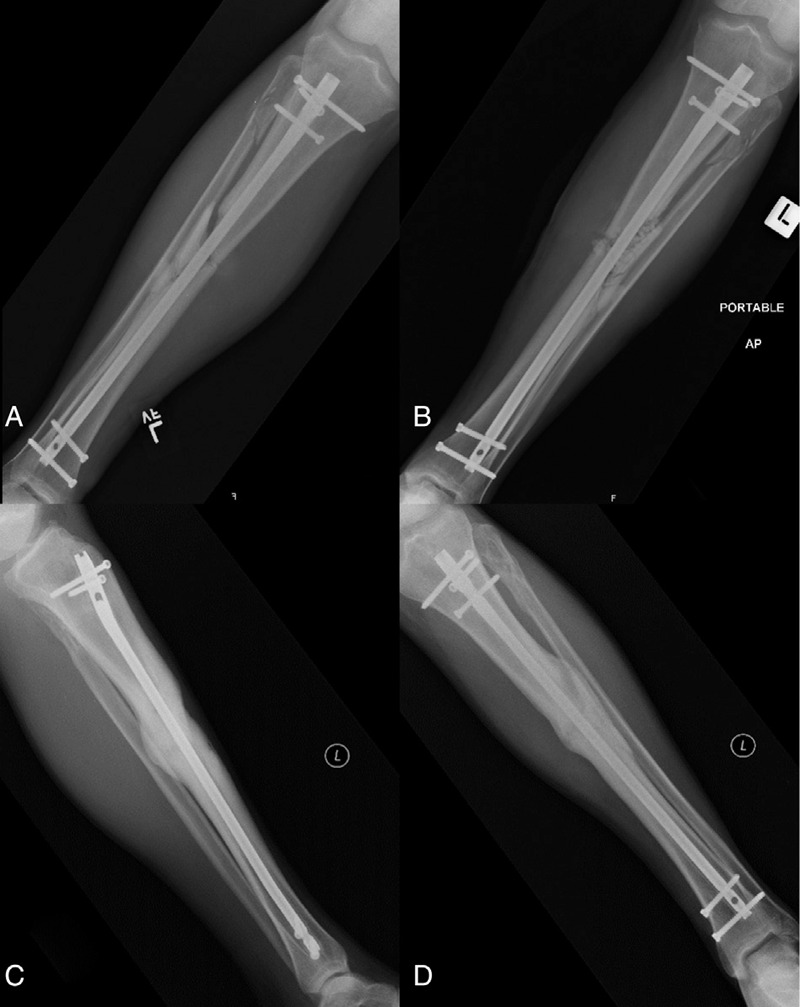
**Acute FRI.** Radiographs of a 39-year-old male who presented with a grade II open left diaphyseal tibia fracture following a motor vehicle accident. He was initially treated with irrigation and debridement, intramedullary nailing, and primary skin closure. He presented 4 weeks postoperatively with increasing pain at the fracture site combined with erythema and wound drainage. Radiographs at that time demonstrated stable hardware (A). He was taken back to the operating room for irrigation and debridement, examination of the hardware for stability, deep tissue cultures, and the placement of local antibiotics including vancomycin powder and antibiotic calcium sulfate beads (Osteoset T, Wright Medical) (B). His original hardware was maintained, and his intraoperative cultures grew Enterococcus Faecalis. He was placed on tailored IV antibiotic therapy for 6 weeks followed by 3 months of oral antibiotics. No further surgical treatment was required and his 1 year follow-up radiographs demonstrate solid union (C and D).

When a peri-implant FRI is suspected, it is important to evaluate the timing of the infection from index surgery. While an acute infection (typically defined as one occurring within 6 weeks of index surgery) can put the patient at risk of developing a nonunion, there is quality evidence that the hardware can be retained in this setting, provided several prerequisites are met.^[20,21]^ First, an adequate debridement should be performed and several deep tissue cultures should be obtained with subsequent targeted antibiotic therapy. Second, the stability of the hardware should be confirmed (both radiographically and at the time of surgery). Finally, the administration of local antibiotics should be considered (see Fig. 1). In a retrospective series of 121 patients with an acute FRI who underwent irrigation and debridement, retention of their original hardware, and antibiotic therapy, Berkes et al^[22]^ reported a 71% success rate of fracture union, with 36% of patients undergoing planned hardware removal after bony union.

The possibility of chronic FRI should be considered in all presenting nonunion cases in the setting of orthopaedic hardware. The rate of reported FRI after fixation varies greatly between less than 1% to greater than 30% depending on a host of important risk factors.^[23,24]^ When evaluating for a potential infectious etiology, one must consider injury factors such as open injury, significant soft tissue damage, contamination, and delayed wound healing; patient factors such as obesity, smoking, malnutrition, diabetes and immunocompromise; and prior treatment factors such as delayed antibiotics, previous external fixation, and long operative time or multiple previous operations.^[25–27]^ All patients presenting with nonunion should be investigated for infection, and the presence of 1 or more of these factors should raise the index of suspicion for infection significantly.

Investigation for FRI in the setting of a nonunion should consist of bloodwork for inflammatory markers including white blood cell count (WBC), erythrocyte sedimentation rate (ESR), and c-reactive protein (CRP). In addition, radiographs should be carefully examined for signs of infection including osteolysis, hardware loosening, and periosteal reaction. Stucken et al^[28]^ investigated a large cohort of patients with a fracture nonunion as well as risk factors for infection and determined WBC, ESR, and CRP provide the best prediction of infection, especially when their results were combined. They found no additional benefit from the inclusion of nuclear studies in diagnosis.^[28]^ However, intraoperative cultures remain the gold standard. A multicentre large cohort study from Olszewski et al^[25]^ demonstrated that 20% of patients with infectious risk factors undergoing revision surgery had surprise positive cultures despite their preoperative work-up being negative for infection. Thus, any patient presenting with a nonunion in the presence of infectious risk factors should be viewed as having an FRI until definitively proven otherwise and should undergo surgical management as outlined below.

**Figure 2:**
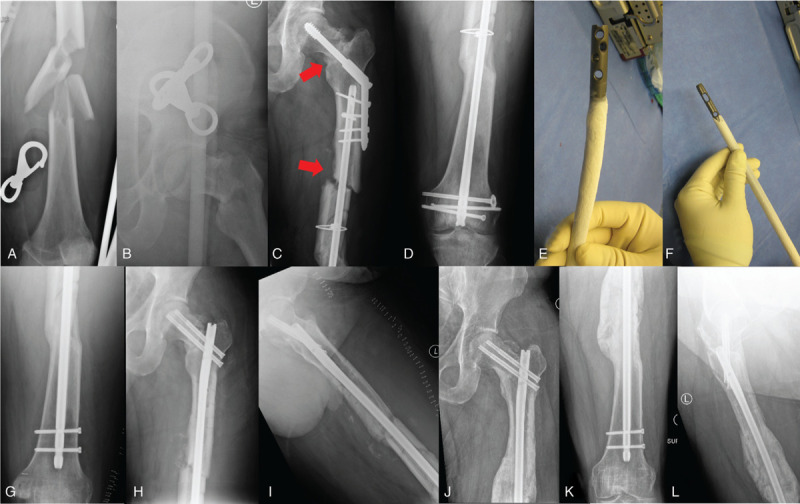
**Femoral neck-shaft combined fracture nonunion.** Radiographs of a 42-year-old male who presented with an open left femoral shaft fracture and closed femoral neck fracture after a motorcycle collision (A and B). He was initially treated with a retrograde intramedullary nail and dynamic hip screw (DHS) construct, which developed a nonunion (C and D). His radiographs demonstrate nonunion of his femoral shaft as well as his femoral neck with varus malalignment of both fractures (red arrows). His preoperative workup revealed normal ESR and CRP and a low vitamin D level, which was corrected prior to surgery. He was taken to the operating room for complete hardware removal, irrigation and debridement, revision fixation with an antibiotic coated cephalomedullary nail (with correction of varus alignment), and placement of platelet-derived growth factor (PDGF, Augment, Wright Medical) at the nonunion site (E–I). Three of 5 deep cultures were “surprise positive” (2/5 for *staphylococcus epidermidis* and 1/5 for *propionibacterium acnes*). He was treated with 6 weeks of postoperative IV antibiotics and 6 weeks of oral antibiotics. Nine-month postoperative radiographs show solid union and the patient was clinically free of infection (J–L).

**Figure 3:**
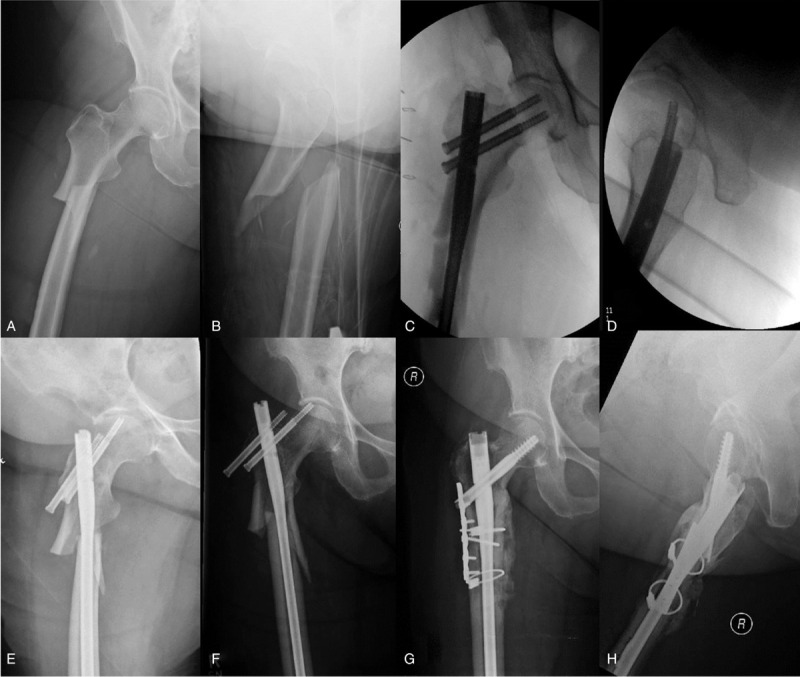
**Atypical femur fracture nonunion.** Radiographs of a 78-year-old female after a low-energy fall demonstrating an atypical femur fracture in the subtrochanteric region (A and B). She had an 8-year history of alendronate use for osteoporosis, and a 3-week history of prodromal right thigh pain prior to fracture. She underwent closed reduction internal fixation with a cephalomedullary nail (C and D). Three-month follow-up radiographs demonstrate progressive varus collapse and hardware failure (E and F). Her ESR was 55 mm/h, and CRP was 13 mg/L. She was taken for revision fixation, including complete hardware removal, irrigation, and debridement with multiple deep tissue cultures. Revision nailing was performed following resection of a small amount of lateral bone and the use of a lateral unicortical plate to correct the previous varus malalignment. Cerclage cables were used to reduce and provide fixation of the large calcar fragment (G and H). Local intrawound vancomycin powder and bone morphogenetic protein (BMP, Infuse, Medtronic) were used to prevent/treat infection and augment biology. Two of the 5 deep intraoperative cultures were found to be positive for *staphylococcus epidermidis*. She received 6 weeks of tailored intravenous antibiotic therapy postoperatively. At 6 months postoperatively, radiographs show solid union and the patient was clinically free of infection.

**Figure 4:**
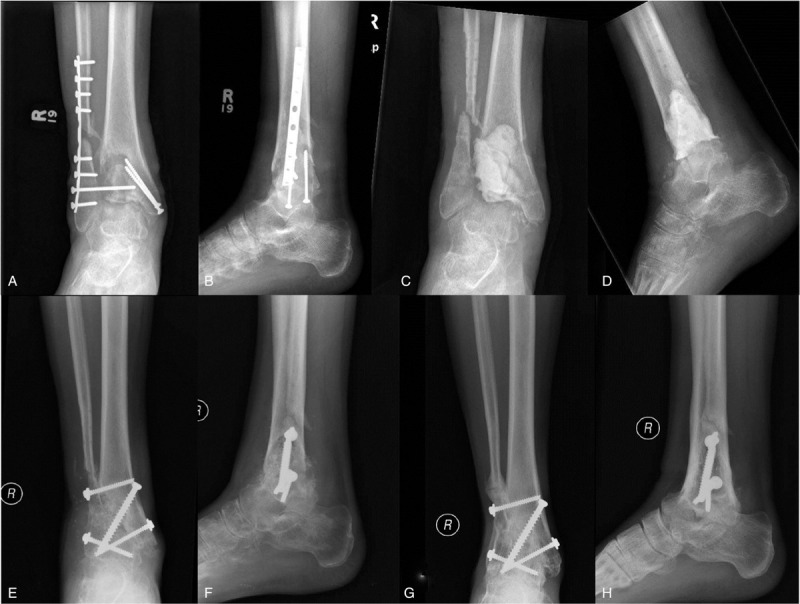
**Infected ankle nonunion and Masquelet technique.** Radiographs of 25-year-old male 6 months after open reduction and internal fixation of a work-related grade II open bimalleolar ankle fracture demonstrating an infected nonunion (A and B). He had ongoing medial wound drainage despite a deep wound washout and prolonged antibiotic therapy. Postoperative radiographs 6 weeks after complete hardware removal, irrigation and debridement, and insertion of an antibiotic cement spacer as the first stage of the induced membrane (Masquelet) technique (C and D). He received 6 weeks of intravenous culture-specific antibiotics and his draining wound healed within 3 weeks of this procedure. Initial postoperative radiographs following removal of the cement spacer and ankle fusion with Iliac Crest bone graft and platelet-derived growth factor (PDGF, Augment, Wright Medical) insertion (E and F). Follow-up radiographs 1 year after the second stage procedure demonstrated solid union of the ankle fusion (G and H). The patient was able to return to work as a landscaper.

When chronic FRI is suspected or confirmed in the setting of nonunion, all of the hardware should be removed, including any broken hardware (see Figs. 2 and 3). A thorough debridement of nonviable or infected tissue and bone should be performed with a minimum of 3 to 5 deep tissue culture samples sent for microbiological analysis. Cultures should be incubated for at least 14 days to avoid missing indolent bacteria such as *propionibacterium acnes* (p. acnes) and *staphylococcus epidermidis* (staph epi).^[29]^ Following this, stabilization of the fracture should be performed. In the setting of gross contamination at the nonunion site, a staged approach can be considered, with intention to return for definitive fixation after a duration of culture-specific antibiotic therapy postoperatively (see Fig. 4). Options for temporary fixation include external fixation, temporary internal fixation, or splinting/casting. It is of utmost importance that stability be maintained in the setting of infectious nonunions, as instability results in soft tissue damage, hematoma, dead-space formation, and impaired revascularization, all of which have been implicated in the pathophysiology of FRIs.^[30]^ In the absence of any gross contamination at the nonunion site, definitive fixation can also be applied after a thorough irrigation and debridement. Special consideration should be given for local antibiotic treatment in the form of antibiotic coated nails, intrawound powdered antibiotics, and antibiotic beads (including commercial absorbable products such as Osteoset T [Wright Medical] and Stimulan [Biocomposites Ltd]). A recent large meta-analysis by Morgenstern et al^[31]^ of open fracture care demonstrated an 11.9% relative risk reduction for development of infection with use of local antibiotic therapy.

Antibiotic cement spacers can also be used in the Masquelet technique to fill bone voids in the setting of extensive debridement or to improve healing potential in the setting of inadequate biology (see Fig. 4).^[32,33]^ This involves a 2-stage reconstruction, with initial insertion of an antibiotic cement spacer in the fracture gap with either accompanying external or internal fixation such as an antibiotic coated intramedullary nail or screw and plate construct for stability, allowing for the formation of a bio-inductive membrane at the site of the critical defect.^[33]^ The second stage is to remove the cement spacer, ideally at 6 to 8 weeks, with careful preservation of the formed membrane and insertion of autologous bone graft and subsequent revision fixation. When applied to tibial shaft defects, the most common site, this is typically performed with an intramedullary nail to provide stable fixation, as well as limit the amount of autologous bone graft that is subsequently required.^[34]^

### 3.2 Loose or failing hardware

The removal of broken or failing hardware can present challenges in the setting of nonunion management. Whenever possible, hardware should be removed to both allow for adequate revision stabilization as well as to eradicate any potential infection that is contributing to the nonunion.

**Figure 5:**
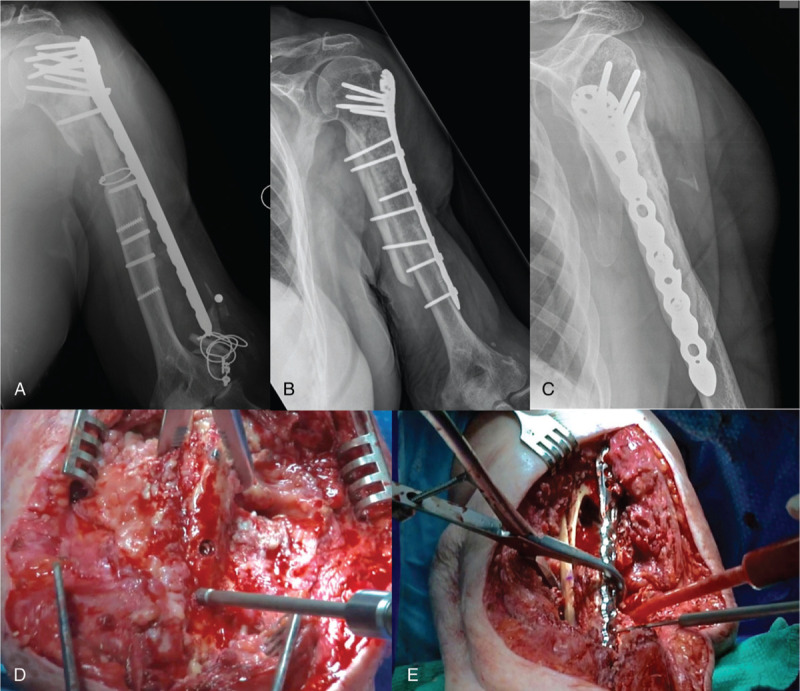
**Proximal humeral nonunion and broken/failing hardware.** Radiographs of an 80-year-old woman with ongoing pain and dysfunction of her left arm following multiple previous surgeries over the past year, demonstrating a proximal humeral nonunion and broken/failing hardware (A). She had no clinical signs of infection. Her infectious bloodwork demonstrated mildly elevated WBC, ESR, and CRP. She underwent revision surgery consisting of complete hardware removal, irrigation, and debridement with deep cultures and revision open reduction internal fixation of her left humerus. The broken screws were removed with a hollow reverse cutting reamer and conical extractor (D). A tibial strut allograft was used to provide additional stability across the nonunion and a proximal humerus locking plate was applied (E). In addition, cancellous morselized allograft and bone morphogenetic protein (BMP, Infuse, Medtronic) were applied at the nonunion/bone defect site. All of her intraoperative cultures were negative at 14 days. Follow-up x-rays at 1 year demonstrate bony union and the final construct (B and C).

Broken or stripped screws and broken intramedullary nails can be very difficult to remove and often require special equipment. In the case of broken screws, often the screw heads are easily removed or loosened, while the distal threaded portion remains in the bone. This is seen frequently with conventional screw and plate constructs and with locking screws for intramedullary nails.^[35]^ In the setting of a healed fracture without concern for infection, it is often most appropriate to leave the screw fragment behind. However, in the setting of a nonunion where exchange nailing is required or if there is concern for associated infection, the entirety of the screw should be removed. A broken screw removal set is indispensable in this setting. For removing the distal fragment of a screw, a reverse-cutting hollow reamer can be used to remove the adjacent cortical bone around the screw. The reamers are available in a variety of sizes and should be selected based on the thread diameter of the screw so as not to make a larger cortical hole than necessary. The screw is then easily removed with a conical extractor of appropriate size (see Fig. 5). Using a hollow reamer, however, may not be possible if the screw fragment remains inside an intramedullary nail hole. In this case, it is easier to push the screw fragment through the opposing cortex by malleting a Steinman pin small enough to fit through the nail hole.^[36,37]^ The broken screw tip can then be retrieved through a separate incision, as necessary. In the setting of a broken cannulated screw, the screw tract should be identified using a free Kirschner wire and the tissue and bone cleared to expose the proximal end of the screw fragment. A conical-shaped reverse threaded extraction tool should then be inserted in line with the screw tract to bind the fragment and remove it.^[38]^

Stripped screw heads are commonly encountered during hardware removal, typically caused by inadequate alignment of the screwdriver with the screwhead prior to turning upon insertion or removal, or by excessively tightening the screw on insertion. In addition, locking titanium screws can become “cold-welded” to the plate, predisposing to screw stripping.^[39–41]^ Many techniques have been described to remove stripped screws including adjuncts such as bone wax, gauze, or foil over the screwdriver head to increase the contact force with the screw driver.^[42]^ Other options include using a conical reverse threaded extraction tool^[35]^ or a high speed metal burr to cut the plate or screw head with subsequent removal of the screw using the broken screw removal set as above. It is critical to plan ahead and have this equipment available.

**Figure 6:**
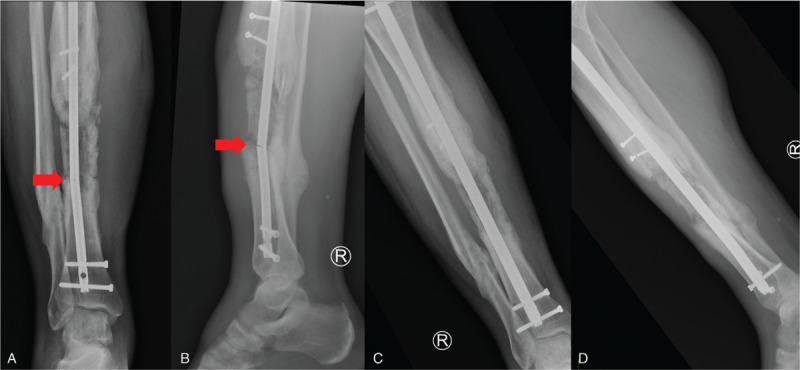
**Tibial shaft nonunion with broken IM nail.** Radiographs of 34-year-old male 6 months after receiving bone grafting of a large bone defect following the induced membrane (Masquelet) technique for an open tibial fracture (A and B). He unfortunately broke his tibial nail prior to his bone graft consolidating fully. Postoperative radiographs removal of the broken nail and exchange to a larger tibial nail (C and D). The broken nail was removed using a commercial extraction hook.

Broken or bent intramedullary nails can prove significantly challenging to remove. In the case of a bent intramedullary nail from secondary trauma, we first attempt to carefully remove the nail in routine fashion. If the bend exceeds what can be removed without other intervention, techniques for removal include attempting to bend back the nail in situ so that it is straight enough for removal, cutting the nail at the apex and removing it in 2 pieces, and/or partially cutting the nail at the apex, followed by attempting to straighten the weakened nail and remove it as a whole.^[43]^ In the case of a broken intramedullary nail, the proximal portion of the nail is often able to be removed in routine fashion. Removal of the distal portion of the nail, however, can prove challenging. There are many described techniques for removing the distal nail fragment including using stacked ball-tipped guidewires, laparoscopic forceps, retrieving the nail from the distal end, etc.^[44]^ Our preference is to use a commercial extraction hook that is placed through the inner canal of the nail, hooks on to the distal end and is then back-slapped to retrieve the nail fragment (see Fig. 6). An alternative, well-described technique used when a commercial extraction hook is not available is to pass a ball-tipped guidewire through canal of the nail. Several other guidewires are then placed in the canal to fill the canal and prevent the ball-tipped wire from pulling out of the nail. A t-handle is then placed on the ball-tipped wire and back-slapped to extract the nail fragment. Both of these techniques often require first over-reaming the proximal canal to allow for easier passage of the broken fragment as it passes proximally during extraction.

### 3.3 Malreduction or malalignment

Malalignment has been demonstrated to be a risk factor for nonunion development, as it can significantly impact the biomechanical stability of the construct. This is particularly evident in proximal and distal femur fractures, where the nonunion rate is reported to be 6% to 10%.^[45–48]^ Riehl et al examined a series of proximal femoral fractures and found any malreduction greater or equal to 10 degrees in either varus or flexion led to either delayed union or nonunion.^[47]^ Similarly, Krappinger et al recently demonstrated varus malalignment of greater than 5 degrees and loss of medial support were both significant risk factors for nonunion.^[49]^ Peschiera et al examined a series of nonunions in distal femoral fractures and found varus alignment of the distal fragment of more than 5 degrees in greater than half of their nonunion cases.^[45]^ This was attributed to medialization of the distal articular block—a common technical error with the use of a distal femoral locking plate.

**Figure 7:**
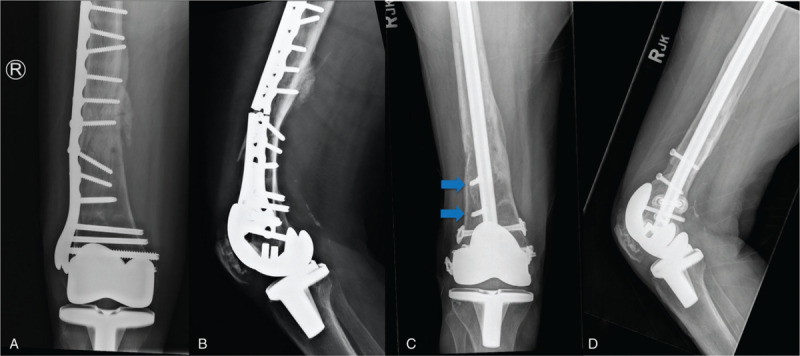
**Periprosthetic distal femoral nonunion.** Radiographs of 68-year-old female 9 months after open reduction and internal fixation of a periprosthetic distal femur fracture, demonstrating nonunion and plate breakage (A and B). The initial construct was overly rigid with too short of a plate, insufficient working length, excessive screw fill, and inadequate spacing of fixation. Postoperative radiographs 6 months after revision fixation using a retrograde intramedullary nail and bone morphogenetic protein (BMP, Infuse, Medtronic) (C and D). Note the use of blocking screws to restore anatomic alignment (blue arrows).

When malalignment is identified as a contributing cause to nonunion, it is critical that this is corrected during revision surgery. This can often require the use of adjunctive techniques such as mini-open reductions, osteotomies, unicortical plating, or blocking screws (see Figs. 3 and 7). Afsari et al examined clamp-assisted, mini-open reduction of subtrochanteric fractures prior to IM nailing and demonstrated a high rate of anatomic reduction and very low rate of nonunion.^[50]^ Additionally, for proximal femur fractures, obtaining correct alignment of the lateral wall has been established as a key to preventing varus malalignment and nonunion.^[51–53]^ In a revision setting for varus or flexion deformity of the proximal femur, the use of a unicortical anterolateral or direct lateral plate or cerclage wires to ensure anatomic reduction can be helpful adjunct prior to repeat cephalomedullary nailing (see Fig. 3). In addition, nail start point often requires medialization to ensure correction of varus. When planning the revision fixation construct, it is important to recognize that additional stability may be required to prevent recurrent malalignment. This can be achieved with the use of additional plating constructs or allograft (see Fig. 5).

### 3.4 Inappropriate construct

Technical error in construct choice or application during initial fixation is another factor that can contribute to the development of nonunion. Often, this is seen when an overly rigid construct is applied in a scenario where secondary bone healing should be targeted.^[54]^ Secondary bone healing requires micromotion and strain across the fracture site to promote callus formation and bony union.^[55,56]^ This approach is particularly useful in diaphyseal fractures of long bones or in comminuted fractures where interfragmentary compression is unlikely to be achieved.^[9]^ The application of an overly rigid construct in these settings can lead to a nonunion, which has become of particular relevance with the introduction of precontoured locking plates.^[48]^ When applied appropriately, locking plates can be extremely useful constructs in fracture fixation, particularly in the setting of significant comminution or osteoporotic bone.^[57]^ However, care must be taken to avoid the creation of an overly rigid construct. The use of inappropriately rigid plates, too many screws, screws across the fracture site, and inadequate plate length have all been demonstrated to increase nonunion rates due to their mechanical effects on construct rigidity.^[58]^ When addressing fractures at particular risk of nonunion with locked plating, such as distal femur fractures with significant comminution or those with a small distal fragment, surgeons should employ the follow principles of fixation: selection of a plate with a working length that is 3 times the length of the fracture/region of comminution or at least 8 holes above the fracture, target a wide spread of screw fixation with less than 50% of diaphyseal screw-hole fill and avoid screw fixation across the fracture site.^[45,57,59]^ Additionally, far cortical locking screws (either by design or technique) have been shown to distribute forces more evenly in locked plating constructs and avoid excessive rigidity of the near cortex, leading to increased callus formation and improved union rates.^[60,61]^ Failure to adhere to these principles can be a common contribution to nonunion, and it is important that any errors in construct selection or application are corrected at the time of revision surgery (see Fig. 7).

## 4 Section 3: managing a nonunion and hardware removal: tips and tricks

### 4.1 Do your homework

Knowledge of the existing implants and previous surgical approaches is of the utmost importance as this will allow planning for the appropriate revision surgery and ensure that the correct removal equipment is available. Riedel et al^[62]^ created a compatibility guide for orthopaedic hardware that is an excellent resource. Preoperative infectious bloodwork and assessment of risk factors for infection will help inform your decision to retain or remove hardware and the need for a debridement.

### 4.2 These are difficult cases, so plan accordingly

Revision trauma cases often require significant operative time, difficult surgical approaches, and the need for multiple implants and equipment. In addition, the implants in situ may be unknown or outdated. Devising a surgical plan that reflects this difficulty and allows adequate time with the correct staff is strongly recommended. It is important to prepare for both expected and unexpected complications. If broken or damaged hardware is a potential, having the appropriate equipment immediately available, such as screw removal sets, Steinman pins, nail extractors and metal cutting burrs, is critical to success. It is often beneficial to have an initial surgical plan that is well thought out, in addition to contingency plans.

### 4.3 Remove all hardware, obtain deep cultures and complete a thorough debridement in the setting of nonunion and potential infection

Up to 20% of patients with risk factors for infection but no clinical signs and negative bloodwork may have positive intraoperative cultures.^[25]^ Cultures should be incubated for at least two weeks to ensure capture of indolent organisms. Adequate debridement, removal of hardware and obtaining cultures to tailor antibiotic therapy will help increase the likelihood of infection eradication and successful nonunion treatment. Furthermore, appropriate stability should be maintained irrespective of whether a single stage revision or staged procedure is planned.

### 4.4 Correct malalignment

As malalignment is a frequent contributor to nonunion, it is important to correct this to increase the likelihood of success with subsequent healing. As outlined above, this can often require the use of ancillary techniques and/or fixation.

### 4.5 Provide additional stability

Consider additional stability using the techniques outlined above in nonunion cases where inadequate mechanical stability or malalignment were contributing factors.

### 4.6 Consider local antibiotics

Infection prevention or the treatment of a potential low-grade infection should be a consideration in all nonunion cases. Delivering local, high dose antibiotics via intra-wound antibiotic powder, antibiotic cement spacers, cement beads or antibiotic coated nails are all potential strategies that should be strongly considered.^[31]^

## 5 Conclusion

Both fracture nonunion and FRI represent substantial sources of pain, impaired function, psychological distress and healthcare costs in orthopaedic trauma. When undertaking nonunion treatment, it is important to recognize the root cause of the nonunion is often multifactorial, and as such, a comprehensive approach to diagnosis, patient optimization and surgical revision must be taken. In the context of nonunion management, hardware removal should be performed in any patient with risk factors or concerns for infection, loose or failing hardware, malreduction/malalignment or inappropriate fracture fixation construct. Following the principles outlined in this article can help to ensure a high rate of success in both FRI and nonunion management.
